# Exploring the Complexity of Children’s Math and Vocabulary Learning: The Role of Cognitive, Dispositional, and Parental Factors

**DOI:** 10.3390/bs15040527

**Published:** 2025-04-14

**Authors:** Zhengqing Li, Keting Chen, Kevin P. Rosales, Jingjing Xu, Lisa Looney, Xin Zhou

**Affiliations:** 1 Morgridge College of Education, University of Denver, Denver, CO 80210, USA; zhengqing.li@du.edu; 2 Department of Child Development, California State University, San Bernardino, CA 92407, USA; kevin.rosales@csusb.edu (K.P.R.); lisa.looney@csusb.edu (L.L.); 3 Shanghai Punan Kindergarten, Shanghai 200135, China; xudindin@gmail.com; 4 Department of Early Childhood Education, East China Normal University, Shanghai 200062, China; xzhou@pie.ecnu.edu.cn

**Keywords:** early mathematics, vocabulary development, attentional control, learning approaches, parental education, kindergarten

## Abstract

Early mathematical and vocabulary skills serve as critical foundations for academic success, yet the mechanisms underlying their development remain complex. This study examines the role of parents’ education, children’s attentional control, and learning approaches as predictors of kindergarteners’ mathematics and vocabulary performance. Using a sample of 149 children aged 60–72 months in Shanghai, China, we conducted a path analysis to explore direct and indirect relationships among these factors. Findings indicate that parental education indirectly predicts math ability through children’s learning approaches and attentional control, emphasizing the role of both cognitive and behavioral pathways. Conversely, vocabulary development is directly influenced by parental education and learning approaches, suggesting distinct developmental trajectories for math and language acquisition. These results highlight the interconnected nature of cognitive, behavioral, and environmental influences on early academic performance. Implications for early childhood education emphasize the need for targeted interventions that not only engage parents in fostering language-rich and cognitively stimulating environments but also support children’s motivation, persistence, and attentional capacities.

## 1. Introduction

The start of a child’s formal academic journey brings with it exposure to a variety of new subjects and teachings. Young children come to the academic space with a level of informal knowledge acquired outside the school setting that is enhanced through formal teaching of concepts and skills in school (Levine et al., 2010; Sénéchal & LeFevre, 2002; Zippert & Rittle-Johnson, 2020). Among the skills learned within and outside the academic environment, mathematics and vocabulary skills often emerge as critical foundational abilities in the educational setting (Jordan et al., 2009; Nation & Snowling, 2004). Learning basic math concepts such as counting, shapes, patterns, and simple operations helps children develop logical reasoning, problem-solving abilities, and number sense (Jordan et al., 2009; Paliwal & Baroody, 2020; Stock et al., 2007). These skills are essential for more advanced mathematical understanding in later grades (Jordan et al., 2009) and for navigating everyday tasks that require quantitative reasoning. Similarly, a strong vocabulary supports language development, comprehension, and effective communication (Dickinson et al., 2003; Hjetland et al., 2019; Topping et al., 2013). A strong vocabulary enables children to express their thoughts, understand instructions, and engage with stories and discussions (Hoff, 2006). Vocabulary growth in kindergarten is also linked to improved reading comprehension and literacy skills (Hjetland et al., 2019), which are critical for learning across all subjects (Hjetland et al., 2019).

Given the importance of early development in math and vocabulary, many research studies have focused on the factors involved in the acquisition of these skills, working to pinpoint influential mechanisms to explain math and vocabulary achievement (e.g., Blums et al., 2017). Specifically, factors like socioeconomic status (Blums et al., 2017; Hoff, 2006), parenting interactions (Hoff, 2006), exposure to books (Combs & Higgins, 2024; Halle et al., 1997; Sénéchal & LeFevre, 2002), classroom curriculum (Cabell et al., 2025; Montague et al., 2014), cognitive abilities (Gunderson et al., 2012; Swanson, 1999; Zheng et al., 2011), and motivation for learning (Liu et al., 2022; Christodoulou et al., 2024; Tsujimoto et al., 2018) have all been examined in relation to early mathematic and vocabulary development, with findings confirming these factors as important predictors of successes and challenges in both math and vocabulary skills.

While connections between various factors and math and vocabulary achievement are worthy of note, the reality is that levels of complexity within and among these factors make understanding influences on early math and vocabulary development much more nuanced. Long-standing theoretical approaches in the field (e.g., Bronfenbrenner, 1979) and advanced understanding of nature-nurture interactions (Scarr & McCartney, 1983) highlight this complexity through explanation of the intersectionality between contexts and between personal characteristics and context as an influence on a variety of educational outcomes (Alloway & Alloway, 2010; Looney et al., 2024; Odom et al., 2004). Thus, the more research can employ examinations of complex mechanisms involved in early achievement, the deeper the understanding of the phenomena present.

When it comes to educational outcomes, research has made clear that complex factors contribute to early and later achievement (Costa et al., 2024). Recently, *Mindset X Context Theory* has guided some of this work to illustrate that educational attainments are a function of both an individual’s personal dispositions and the contextual environments that foster those personal characteristics (Carroll et al., 2023; Walton & Yeager, 2020). That is, the psychological resources one brings to an educational setting (e.g., intrinsic interest, persistence, general motivation) can be enriched or hindered based on contextual influences (e.g., opportunities or constraints) that might be present (Carroll et al., 2023; Miller & Maricle, 2019; Walton & Yeager, 2020). A young child with an inherent interest in numbers or words, for example, might be able to foster that interest in a setting that contains opportunities for conversation, toys that develop numeracy, and exposure to books and other educational materials. Conversely, without those contextual opportunities available, interest in those areas might not be realized. Therefore, exploring both personal attributes and context becomes critical for understanding achievement.

Adding to the complexity of influences on academic success is the role of cognitive processes. Research evidence has shown that cognitive abilities enable students to process, store, and apply information effectively (Baddeley, 2007). Specifically, executive function, working memory, processing speed, cognitive flexibility, and attention facilitate a students’ ability to engage in and sustain tasks that are often correlated with positive academic outcomes (Alloway & Alloway, 2010; Spiegel et al., 2021; Zelazo & Carlson, 2020). That is, when students can plan and problem solve, hold and manipulate information, process information efficiently and accurately, adjust to different educational settings, and attend to academic tasks while filtering out distractions, the more likely they are to accomplish positive academic outcomes (Alloway et al., 2009; Gathercole et al., 2008; Simone et al., 2018). With regard to math and vocabulary skills, numerous research studies have shown a significant positive relationship between these cognitive abilities and math and vocabulary achievement (Noël, 2009; Peng et al., 2016; Peng et al., 2018).

In short, the message that results from these various research findings and theoretical foundations is that the factors that predict math and vocabulary ability are many. While there are studies that employ models and statistical techniques to capture some of this complexity, research is often segmented into various camps. One might argue, for example, that in the extant literature, contextual complexities (e.g., SES, parental involvement, teacher characteristics, environmental stimulation) are studied in tandem with either psychological complexities (e.g., motivation, personality attributes) or with cognitive complexities (e.g., attention, working memory, processing speed). What is more rarely seen (if seen at all) is a linkage of all three areas of complexity, as cognitive and more social-emotional factors (e.g., motivation and personality characteristics that guide the formation of learning approaches) are often viewed as distinct elements and are seldom examined together.

Increasing our understanding of how context, personal social-emotional attributes, and cognitive components work together to influence math and vocabulary skills is warranted. Discussions of complexity within various strands of literature center on such topics as the domain-general or context-dependent nature of cognitive abilities (Doebel & Müller, 2023; Hughes, 2023; Niebaum & Munakata, 2023), the value of person-oriented over variable-oriented motivational research (Spiegel et al., 2021; Viljaranta et al., 2016), and the high degree of connectivity of cognitive and emotional regions of the brain (Pessoa, 2008). Each of these discussions draws attention to the intersectionality of various individual and contextual components to explain how one might excel or struggle in academic settings.

### 1.1. The Intersection of Personal Attributes, Cognition, and Context

Individuals’ approaches to learning, including motivational qualities (Christodoulou et al., 2024; Viljaranta et al., 2016), personality or temperament traits (Christodoulou et al., 2024), and cognitive abilities (Alloway & Alloway, 2010; Gathercole et al., 2008; Simone et al., 2018), are among some of the characteristics that are related to how a child fares within the academic setting. These psychological orientations and cognitive capacities contribute to a child’s ability to engage with classroom tasks and activities, attend to relevant academic material to enhance learning, persevere through challenging tasks or setbacks, and engage in planning strategies that help to solve problems or complete academic tasks. Research has demonstrated that these resources play an important role in whether a student succeeds or struggles in the educational setting (Christodoulou et al., 2024; Gathercole et al., 2008; Viljaranta et al., 2016). However, contextual factors such as SES, access to resources, parental education levels, school environments, and family support influence both personal attributes and cognitive development (Blums et al., 2017). That is, children from low-SES backgrounds might face stressors that impair their ability to attend to learning or enhance their ability to recognize challenges and rise above them, whereas those in resource-rich environments might benefit from tools that enhance learning and parents that view education as valuable, demonstrating positive attitudes toward learning. Together, personal attributes, cognition, and context create a dynamic interplay of various patterns of development that shape academic outcomes (Carroll et al., 2023).

### 1.2. The Current Study

With the need to understand how context, personal learning approaches, and cognition work together to contribute to academic outcomes, the current study aimed to examine these elements in relation to children’s early math and vocabulary achievement. Specifically, with the use of path analysis, the current study assessed the direct and indirect impacts of parental education (context), children’s approaches to learning (personal psychological attributes), and attentional control (cognition) on early mathematics and vocabulary achievement.

*Parental level of education.* The relationship between the parental level of education and children’s academic achievements is a much-studied topic in the literature, demonstrating that how far parents go in their own schooling can significantly influence children’s math and vocabulary development through mechanisms tied to the home environment, parental involvement and beliefs, and provisions of resources afforded to children (Blums et al., 2017; Davis-Kean, 2005; Halle et al., 1997; Tan, 2015). For example, educated parents are more likely to engage in conversations with their children that include a richer and more complex vocabulary (Olaru et al., 2022; Rowe, 2008), exposing children to more advanced language from an early age. Similarly, these parents often incorporate activities like counting and number games into daily routines, thereby fostering early numeracy skills (Douglas & Rittle-Johnson, 2024). Access to resources might also be enhanced for children with educated parents, as higher education is often associated with greater financial stability, allowing for parents to provide educational toys (e.g., books, puzzles) and technology that might further enhance math and vocabulary capabilities (Davis-Kean, 2005; Halle et al., 1997).

Higher levels of parental education are also associated with parenting practices also found to factor into children’s academic achievement. For instance, studies have found that mothers with higher educational attainment are more likely to actively participate in their children’s education by reading to their children more regularly (Yarosz & Barnett, 2001), helping them with math and reading homework (Fantuzzo et al., 2000), or taking an active role in the school environment (e.g., attending school events, helping out with classroom activities, communicating with teachers), all of which is important for children’s academic achievement (Burchinal et al., 2002; Davis-Kean, 2005; El Nokali et al., 2010).

*Learning Approaches.* Children’s approaches to learning are varied and complex. Factors such as interest in a topic area, initiative to seek out learning opportunities, value of the task, perseverance through difficult tasks, and awareness of task goals act as drivers of engagement, effort, and motivation in academic tasks (Anthony & Ogg, 2019; Eccles & Wigfield, 2020; Viljaranta et al., 2016). Research in these areas tends to focus on a specific aspect of a learning approach (e.g., interest in a task *or* perseverance) rather than viewing them as a pattern of a child’s learning characteristics. For instance, studies have demonstrated that when children are interested in a topic, they are more likely to explore it on a deeper level, ask more questions related to the topic, and seek out information about the topic, all of which can contribute to increased achievement in the topic area (Christodoulou et al., 2024; Fung et al., 2018; Ma, 2022). Similarly, because the academic environment often presents various challenges (e.g., complex tasks), children who demonstrate perseverance—particularly with regard to math or vocabulary—are more likely to achieve academic success relative to counterparts who might give up easily (Fung et al., 2018; Zhang et al., 2024). Therefore, on their own, various approaches to learning have a clear link to academic achievement. However, more recent research (Viljaranta et al., 2016) has highlighted the importance of examining patterns of learning, as various profiles can perhaps demonstrate different relationships to achievement (Christodoulou et al., 2024; Ma, 2022; Viljaranta et al., 2016). That is, examining how certain approaches to learning hang together in a particular profile might help to further highlight the complexity of person-oriented approaches to learning and their relationship to math and vocabulary achievement.

*Attentional control.* Controlling attention on a task requires one to inhibit a focus on distractors (Engle, 2002). When children can focus attention, sustain attention on a given task, manage distractions, and shift attention when needed, this level of control can play a role in the successful completion of academic tasks such as math and vocabulary (Isbell et al., 2018; Orbach & Fritz, 2022; Sánchez-Pérez et al., 2015). Specifically, a greater ability to control attention when engaged in math and vocabulary tasks allows for greater processing of information related to academic assignments (Isbell et al., 2018; Li et al., 2023), a higher likelihood of understanding mathematical patterns and new words (Carretti et al., 2009; Li et al., 2023), and an enhancement of comprehension and recall (Carretti et al., 2009). Being able to shift attention allows children to shift focus to different learning or problem-solving strategies when challenges occur (Isbell et al., 2018; Li et al., 2023), and an ability to reduce distractions helps children to attend to relevant instructional information and concentrate on relevant aspects of a learning problem (e.g., pronunciation or comprehension of a word, solving a math problem), without being disrupted with irrelevant information (Isbell et al., 2018).

**Figure 1:**
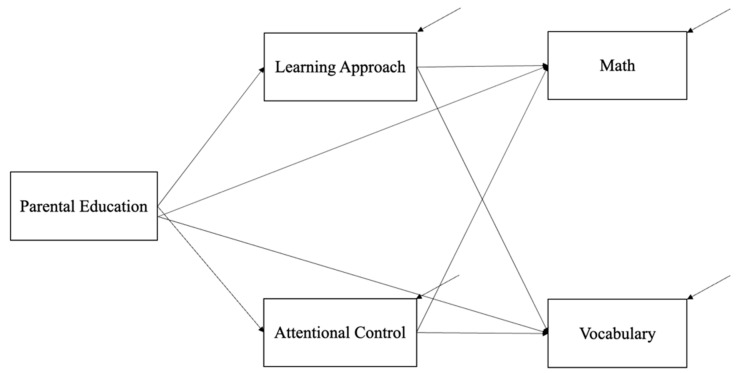
Conceptual mediation model.

*Parental education, learning approaches, and attentional control.* As has been previously stated, each of these variables has been established as important for academic achievement but has rarely (if at all) been studied together. Given what we know about the complexity of factors associated with children’s ability to achieve in subject areas, the current study sought to examine how context (parental education), learning approaches (personal attributes), and attentional control (cognition) work together to impact children’s math and vocabulary ability (see Figure 1). Specifically, we utilized contextual and ecological theoretical models (e.g., Bronfenbrenner’s Ecological Model, Mindset X Context Theory) to examine pathways to illustrate how more distal influences (like parental education) exert their effects indirectly through more proximal processes. Parental education represents a contextual factor that can significantly shape the resources, opportunities, and stressors children encounter. These environmental influences can affect children’s learning approaches and cognitive processes which, in turn, impact their academic outcomes. Thus, our conceptual mediation model seeks to capture the dynamic, multi-level interactions described by theoretical frameworks, offering a more holistic and complex understanding of how these various factors operate together to impact vocabulary and math performance.

To that end, the following hypotheses were tested:

**H_1_:**  *Parental education has an indirect effect on children’s math ability and children’s vocabulary via children’s learning approaches and attentional control.*

**H_2_:**  *Parental education has a direct effect on children’s math ability and vocabulary.*

**H_3_:**  *Children’s learning approaches and attentional control mediate the effect of parental education on academic performance.*

## 2. Method

### 2.1. Participants

One hundred and forty-nine children and their families were randomly selected from 60- to 72-month-old classrooms in Shanghai, China (M = 67.44 months, SD = 3.73). Among the 149 child participants, 86 were boys and 63 were girls. All participating children were monolingual (in Mandarin) and of Han ethnicity.

### 2.2. Procedure

The current study is a cross-sectional study. The procedure followed the protocol approved by the University Institutional Review Board. The parents of participants gave their consent for themselves and their children’s participation and filled out the survey questionnaire, which included parental education information. Teachers of participants agreed to fill out a test questionnaire about participants. Two graduate students were trained to administer the measures. Participating children were assessed in a quiet room in their kindergarten.

### 2.3. Measures

Three measures were used to assess participants’ attention score, math ability, and vocabulary ability. Each participant completed all three tests within one session.

*The child ANT*. The Child Attention Network Test (ANT) assesses attentional processes in children, which is a test developed on the E-prime platform (Ruead et al., 2004). Children are presented with visual stimuli (e.g., arrows or fish) and respond based on specific task instructions. Research indicates that children perform best in tests with story contexts and outcome feedback (Berger et al., 2000). Therefore, in the child version of ANT, colorful fish are used instead of arrows in the Flanker task. The experimenter invites the children to help feed the middle fish, instructing them to press the corresponding directional button based on the direction in which the middle fish swims. Participants were required to respond to all stimuli that were displayed on a computer screen by clicking the mouse. Each trial began with a central fixation cross. The target array was a yellow-colored line drawing of either a single yellow fish or a horizontal row of five yellow fish, presented above or below fixation, over a blue-green background. The participant was to respond based on whether the central fish was pointing to the left or right by pressing the corresponding left or right key on the mouse. On congruent trials the flanking fish were pointing in the same direction, on incongruent trials the flankers pointed in the opposite direction from the central fish, and on neutral trials the central fish appeared alone (Fan et al., 2002). All the participants were regularly exposed to technology in their kindergarten classroom and thus were familiar with computers and mouse-clicking. Participants had a practice session for about 3 min, within which they received the experimenter’s response and encouragement. Following the practice session, the formal experiment began, and the children no longer received feedback from the experimenter. The attentional control score was computed as the participant’s median reaction time for each flanker condition (across cue conditions) and subtracted the congruent from the incongruent reaction time. Thus, a small or negative attentional control score suggests the child is better at handling interference. The attentional control had the highest test-retest reliability (*r* = 0.77) among the three attention networks (Fan et al., 2002).

*Test of Early Mathematics Ability—Third Edition* (TEMA-3; Ginsburg & Baroody, 2003). The TEMA-3 is a standardized test designed to measure mathematical ability in children between 3 years 0 months and 8 years 11 months. The test was composed of 72 items measuring informal and formal knowledge of both concepts and skills in a variety of domains (Ginsburg & Baroody, 2003). Informal mathematical knowledge is acquired outside the context of schooling, and it underlies the basic mathematical knowledge that is taught in school, whereas formal mathematical knowledge represents the concepts and skills that children learn in school (Ginsburg & Baroody, 2003). Each participant’s binary answers (pass/fail) were recorded on a form. This test has internal consistency alphas equal to or above 0.94 for the different age intervals (Ginsburg & Baroody, 2003). The Chinese version of the TEMA-3 demonstrated good reliability and validity. Specifically, the internal consistency was high, with a Cronbach’s α coefficient of 0.932, indicating strong cross-indicator consistency. The split-half reliability reached 0.747, reflecting satisfactory internal consistency across test items. Additionally, the test-retest reliability was 0.845, suggesting strong measurement stability over time (Kang et al., 2014). For this study, the raw score on the TEMA-3 was used as the math outcome.

*The Peabody Picture Vocabulary Test—Revised Edition* (PPVT-R). PPVT-R is the Chinese version adapted from PPVT-IV, measuring receptive vocabulary skills (Dunn & Dunn, 1997). The PPVT-IV demonstrates good reliability and validity (Wing-Yin Chow & McBride-Change, 2003; Dunn & Dunn, 1997). Lu and Liu (1998) developed the PPVT-R and reported split-half reliability ranging from 0.90 to 0.97, indicating excellent internal consistency among the test items. The participant was shown a card with four pictures, and the assessor read a word and asked the child to point to the picture that corresponds to the word. A standardized score was computed that reflected the extent to which each child’s performance compared to the expected performance of same-age children in the norming population.

*Parental Education.* Parental education was scored as the highest education degree from parents (Santelli et al., 2000). Parental education was scored as 1 = junior school and below, 2 = high school, 3 = associate degree, and 4 = bachelor’s degree and above.

*Learning approach observational assessment.* Learning approach observational assessment is a checklist developed and adapted by a group of Chinese researchers aimed at assessing 10 aspects of children’s learning, including interest, initiation, concentration, persistence, resistance, information use, reflection and explanation, exploration, goals, and independence (Xu et al., 2016). Each aspect has five levels of performance, with specific behavioral descriptions corresponding to each level. Level 5 represents the highest performance, while Level 1 represents the lowest. The internal consistency of the checklist was adequate (Cronbach’s alpha α = 0.94). The internal consistency of each aspect was greater than 0.93. To further test the reliability of this measure, considering that the reporters of this measure were the teachers, a random kindergarten teacher was invited to observe children’s behavior and rate it using this measurement alongside the researcher but separately. The Kendall’s Coefficient of Concordance (W) between the two raters was 0.828, indicating a high level of reliability.

Data were analyzed first using SEM techniques and Mplus 8 software (Muthen & Muthen, 2011) with a bootstrap approach (Shrout & Bolger, 2002). To assess the adequacy of our sample size, a post hoc power analysis was conducted. With N = 149 and α = 0.05, the power to detect large effects (f^2^ = 0.35) was 98.9%. Thus, the study is sufficiently powered for large effects. Bootstrapping was used to provide a practical approximation of sampling distributions of indirect effects to produce confidence intervals (CI) of estimates. An indirect effect is different from zero when the confidence interval does not contain zero. In the current study, we performed a nonparametric resampling method with 1000 resamples drawn to derive the 95% CIs for the indirect effect of parental education on math and on vocabulary through learning approach and executive attention. Multiple indices, including the Comparative Fit Index and Tucker Lewis Index (CFI & TLI; Bentler, 1990), the Root Mean Square Error of Approximation (RMSEA; Browne & Cudeck, 1993), and the Standard Root Mean Residual (SRMR; Hu & Bentler, 1999), were used to assess global model fit. To determine good model–data fit, recommended cutoffs are >0.90 for the CFI and TLI and <0.10 for the RMSEA and SRMR (Kline, 2011; Mueller & Hancock, 2010). Data analyses were conducted in two phases. First, preliminary analyses were conducted, and descriptive statistics were obtained to determine whether the data met the basic assumptions of SEM. The second phase of data analysis was to test the mediation path model (see Figure 1).

## 3. Results

### 3.1. Descriptive Statistics

The descriptive statistics and bivariate relationships among variables were analyzed by SPSS 25. The estimates are reported in Table 1. All statistical assumptions were met. There are significant correlations among variables. Attentional control (for which higher levels indicate an increased time to process incongruent situations) negatively correlated to all the other variables, indicating that the higher attentional control scores were associated with lower levels of parental education (*r* = −0.217, *p* < 0.01) and lower scores for learning approach (*r* = −0.196, *p* < 0.05), math (*r* = −0.291, *p* < 0.01), and vocabulary (*r* = −0.221, *p* < 0.01). The learning approach is highly positively correlated to math ability (*r* = 0.82, *p* < 0.01). However, the learning approach was measured by a more domain-general observational tool, while math was measured by a more domain-specific (math content) performing the task. They are considered as two distinct measurements. There were no concerns about the multicollinearity (*r*s < 0.70) of distinct predictors within the model.

### 3.2. Path Model

The path model was tested by Mplus 8 with a bootstrap approach. The “full information” method that estimates all parameters simultaneously, also known as maximum likelihood (ML) estimation, was used for this non-recursive path model as a default. Estimation of this model is a just-identified model with a perfect global fit, CFI = 1.000, TLI = 1.000, RMSEA = 0.000, and SRMR = 0.000. Unstandardized parameter estimates and 95% confidence intervals are shown in Table 2. This path model explained approximately 70% variance in math, 24.9% variance in vocabulary, 21.4% variance in learning approach, and only 4.5% variance in attentional control.

**Figure 2:**
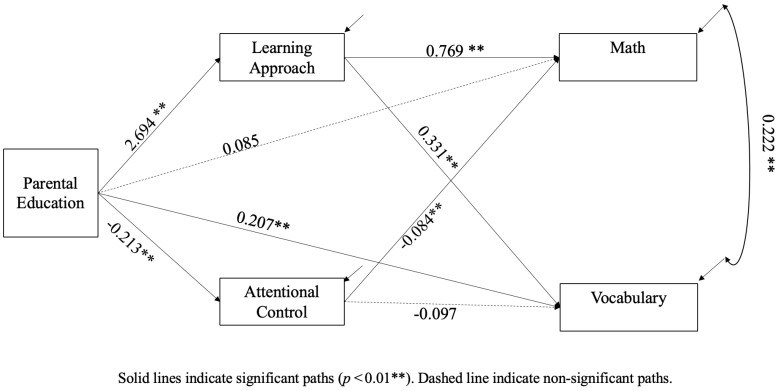
Tested model with standardized estimates.

Tested model with standardized estimates are reported in Figure 2. The 95%CI [2.006, 4.317] for the indirect effect of parental education level on children’ math ability via learning approach does not contain zero. Thus, parental education level impacts children’s math ability by fostering their learning approach. Simultaneously, the 95%CI [0.005, 0.503] for the indirect effect of parental education level on children’s math ability via attentional control does not contain zero, either. However, the 95%CI [−0.240, 1.630] for the direct effect of parental education level on children’ math ability does contain a zero, which means that there is no direct impact from parental education level on children’s math ability. With regard to children’s vocabulary ability, parental education level has a direct impact on vocabulary and an indirect path through learning approaches rather than attentional control. There is no co-variance between the learning approach and attentional control, but there is a co-variance between math and vocabulary within this path model.

To assess the robustness of our findings, we conducted an additional linear regression predicting math performance while controlling for vocabulary ability. The results indicated that vocabulary (*b* = 0.19, *p* < 0.001) and learning approach (*b* = 3.20, *p* < 0.001) were significant predictors of math performance, while attentional control was not (*p* = 0.581). Parental education remained a significant predictor (*b* = 2.56, *p* = 0.011). The model accounted for approximately 39% of the variance in math scores (*R*^2^ = 0.389), supporting the robustness of vocabulary and learning approach in early academic development.

## 4. Discussion

The goal of the present study was to test the complexity of factors (contextual, personal-psychological attributes, and cognitive) that impact math and vocabulary indices. Specifically, we implemented path analysis to examine the direct and indirect effects of parental education, attentional control, and learning approaches on math and vocabulary measures among children. As hypothesized, we provide evidence that these variables impact at least one of the academic outcomes (i.e., math & vocabulary).

An important main finding in the current study is that parents’ level of education indirectly predicted math ability through a learning approach. This corroborates previous research showing that math development is best explained by a complex set of factors as opposed to being determined by one sole influencer (Costa et al., 2024). In this case, math ability was greatly impacted by one’s learning approach, which was demonstrated by numerous traits, including interests, initiation, concentration, persistence, resistance, use of reflection and explanation, exploration, goals, and independence. This finding aligns with numerous research studies that have found connections between these various personality and motivational factors and math and vocabulary achievement (e.g., Christodoulou et al., 2024; Ma, 2022; Zhang et al., 2024) and further corroborates work that examines these psychological orientations as patterns or profiles (rather than one single trait) that might show variability among learners (e.g., Anthony & Ogg, 2019; Viljaranta et al., 2016). Further, our results demonstrated that the traits that define one’s learning approach were influenced by the home environment, measured by parents’ level of education. That is, as theory and research have argued, characteristics found in various learning approaches intersect and can be enhanced or hindered by the contexts in which children find themselves (Carroll et al., 2023). In this case, parents with higher levels of education might have created environments in which these learning approaches could be enriched. Such findings align with the understanding that children’s math performance is a multidetermined construct impacted by both contextual and personal psychological factors. These findings fit well with prominent systems models of development (e.g., Bronfenbrenner, 1979).

Another important finding from the current study is that parental education negatively predicted children’s attention control. Specifically, higher levels of parent education were associated with faster performance on the child attention network test. This suggests that the parents’ level of education may be associated with parents setting up a home environment that is conducive to fostering cognitive abilities, such as access to books, play activities, and other instructionally oriented pieces that have been shown to spark healthy cognitive functioning in children (Sakib, 2022). Further, parents’ level of education also indirectly impacted math ability through children’s attention control. This is a notable finding suggesting that children’s math ability is not only dependent on one’s learning approach but also the child’s cognitive abilities, both of which are directly impacted by parent’s level of education. This set of findings continues to add to the broader theme that child outcomes are best explained through a complex lens.

For children’s vocabulary, the findings show that parental education had a direct impact. Years of language development research can serve to explain this finding. It is well documented that children’s language starts to develop in utero months before a child is born (Mariani et al., 2023). Fetuses listen to the mother’s voice from early on, and newborns can recognize all syllables in the languages spoken to them. Following birth, children are exposed to a myriad of experiences that bolster this language development. Parental interactions, exposure to language-rich environments, and engagement with media all contribute to children’s language development, with parents serving as the primary source of these experiences. Given the strong association between parental education and the richness of linguistic input (Olaru et al., 2022; Rowe, 2008), as well as the provision of educational resources (Davis-Kean, 2005; Halle et al., 1997), it is reasonable to expect that parental education would directly influence children’s language development beyond other contributing factors.

Similar to the math-related findings, learning approaches were shown to be important dispositions to vocabulary development. Learning dispositions, encompassing traits such as persistence, exploration, and goal setting, significantly predicted vocabulary performance in the current sample. These findings align with research suggesting that positive learning behaviors facilitate cognitive engagement and exposure to language-rich experiences, which are essential for vocabulary acquisition (Sénéchal & LeFevre, 2002). Dispositions such as curiosity and reflection may promote active learning opportunities, where children are more likely to seek out, process, and retain new vocabulary (Claxton & Carr, 2004). Thus, the positive relationship between learning dispositions and vocabulary highlights the importance of fostering learning-oriented behaviors that support language development during early childhood.

Taken together, the current results show that the paths to foster math and vocabulary are different. Parental education was shown to indirectly impact math skills through learning approaches and executive functions, while vocabulary was directly impacted by parental education as well as learning approaches. This offers important insight for understanding the developmental trajectories of these academic outcomes and the complexity of the factors that impact them. The differential pathways through which parental education influences math and vocabulary performance provide compelling insights into the nuanced mechanisms driving early academic outcomes. For math, the mediation through learning approaches and executive functions suggests that parental education fosters higher-order cognitive processes and behaviors, such as persistence (part of learning approaches) and attention control, which in turn support math performance (Blair & Razza, 2007; Ursache et al., 2012). This aligns with evidence highlighting the complex nature of math as requiring both behavioral regulation and domain-specific learning characteristics. In contrast, vocabulary development was directly influenced by parental education and learning approaches, underscoring the foundational role of enriched linguistic environments provided by more educated parents (Hoff, 2006; Hart & Risley, 1995). The absence of mediation in vocabulary suggests that exposure to language and rich verbal interactions in the household may be sufficient to shape children’s language outcomes without the heavy reliance on intermediary cognitive processes. These findings reveal the critical role of parents not only in cultivating language-rich interactions but also in fostering dispositions and core cognitive abilities that are instrumental for broader academic success.

There are a few shortcomings of the present study worth highlighting. First, the construct of the learning approach studied here is a conglomerate of several learning characteristics. By nature, this diminishes the construct specificity of this variable. In other words, it is difficult to pinpoint which of the components of the learning approach is most or least influential to the set of variables in this study. While recent research has highlighted the value of studying patterns of learning profiles (Christodoulou et al., 2024; Ma, 2022; Viljaranta et al., 2016), studies following up this work would also benefit from a more refined measure of learning approach, as understanding these variables individually and as a pattern is beneficial. Although parental education level is often used as a proxy for SES, we acknowledge that more granular SES indicators—such as exact years of schooling or household income—could offer improved precision. Our focus was strictly on parental education (and, therefore, these SES indicators were not collected in the current study), but robust SES data would strengthen future research in examining how these contextual elements impact the relationship between these variables. Third, a consideration of the current study is that the path model was just identified, which precludes the use of global fit indices to evaluate model adequacy. While this was intentional—given our goal to test a specific, theory-driven structure—future research could explore alternative or nested models to further assess the robustness and generalizability of the proposed relationships. Last, while path analysis offers structural insight into understanding the complex nature of children’s academic outcomes and the context in which they develop, future work should attempt to explore other analyses like structural equation modeling or network analysis that lend themselves well to examining broad complex developmental structures.

Future studies in this vein of work should attempt to address these questions longitudinally to better understand the developmental trajectory of parental education, learning approach, attention control, math ability, and vocabulary. In addition, prospective studies should explore subgroup analyses (e.g., gender and age) to examine how these effects might vary across stratified subgroups, as this would provide more nuanced information regarding the influences on math ability across demographics. Further, there are a myriad of other variables germane to math and vocabulary, for example, school influences and peer relationships, that would be worthy of study. Moreover, this set of variables would be critical to study within the school context to determine how the role of teachers may affect these outcomes.

Overall, the findings from this study illustrate the complex interplay between parental education, children’s learning approaches, and attentional control in molding early academic outcomes, putting forth a multifaceted understanding of how these factors collectively influence math and vocabulary achievement. Consistent with prior research, parental education emerged as a foundational context, directly supporting vocabulary development through enriched language environments while indirectly influencing math performance via the mediation of learning approaches and attentional control. These results affirm the importance of examining academic achievement as a multidetermined construct, where context, personal dispositions, and cognitive processes converge to influence developmental trajectories. Importantly, this study highlights the unique mechanisms underlying math and vocabulary performance, accentuating the need for targeted interventions that not only engage parents in fostering language-rich and cognitively stimulating environments but also support children’s motivation, persistence, and attentional capacities. By emphasizing these integrated pathways, this work advances the field’s understanding of early academic success and underscores the critical role of parents in cultivating both the cognitive and behavioral foundations necessary for long-term achievement. These insights provide a framework for future research and practice aimed at optimizing children’s academic outcomes in diverse contexts.

## Figures and Tables

**Table 1:** Descriptive statistics and pairwise correlations.

	Mean (SD)	N	1	2	3	4	5
1. Parental Education	3.99 (1.24)	149	-				
2. Attentional Control	2.90 (3.49)	149	−0.217 **	−			
3. Learning Approach	33.77 (7.15)	128	0.454 **	−0.196 *	-		
4. Math	38.00 (10.69)	149	0.456 **	−0.291 **	0.821 **	-	
5. Vocabulary	54.24 (18.79)	149	0.375 **	−0.221 **	0.442 **	0.502 **	-

*p* < 0.05 *, *p* < 0.01 **.

**Table 2:** Unstandardized parameter estimates (and SEs) and confidence intervals.

		Unstandardized			95%CI	
		Estimate	*SE*	*p*-Value	Lower 2.5%	Upper 2.5%
**Direct path**						
Parental education	Math	0.731	0.463	0.114	−0.240	1.630
	Vocabulary	3.138	1.227	0.011	0.770	5.438
	Learning approach	2.694	0.436	0.000	1.849	3.583
	Attentional control	−0.599	0.281	0.033	−1.169	−0.023
Learning approach	Math	1.138	0.091	0.000	0.961	1.319
	Vocabulary	0.860	0.236	0.000	0.407	1.337
Attentional control	Math	−0.257	0.124	0.039	−0.526	−0.008
	Vocabulary	−0.520	0.408	0.202	−1.318	0.306
**Indirect path**						
Parental education—Learning approach	Math	3.066	0.573	0.000	2.006	4.317
	Vocabulary	2.318	0.730	0.002	1.165	4.092
Parental education—Attentional control	Math	0.154	0.112	0.171	0.005	0.503
	Vocabulary	0.312	0.298	0.295	−0.096	1.178
**R-square**						
	Math	0.700	0.047	0.000		
	Vocabulary	0.249	0.058	0.000		
	Learning approach	0.214	0.060	0.000		
	Attentional control	0.045	0.046	0.319		

## Data Availability

Data are contained within the article.
